# Awareness of gingival retraction before final impression procedures among interns at Saudi Arabia: A cross-sectional study

**DOI:** 10.6026/973206300222267

**Published:** 2026-04-30

**Authors:** Rawan Alrethia, Ibrahim AlBaqami, Bayan Almohaimeed, Abdulaziz Alharbi, Mohammed Alqahtani, Abdullah Alhablen

**Affiliations:** 1 Department of Prosthetic Dental Sciences, College of Dentistry, Qassim University, Saudi Arabia; 2 Intern, College of Dentistry, Qassim University, Saudi Arabia; 3 Department of Community Dentistry and Oral Epidemiology, College of Dentistry, Qassim University, Saudi Arabia

**Keywords:** Gingival displacement, fixed prosthodontics, impression

## Abstract

Mechanical gingival retraction using a plain retraction cord has been regularly practiced before impression making in fixed Prosthodontics. Therefore, it is of interest to assess the knowledge and awareness of a variety of gingival displacement methods before impression making in fixed Prosthodontics among dental students. A questionnaire-based survey was administered to dental students and interns at Qassim University. Participants completed a questionnaire concerning their awareness and knowledge of gingival displacement techniques, including the reasons for use and adverse effects and complications. Most participants recognized the importance of gingival retraction in clinical practice but were unaware of its potential complications and adverse effects. Comprehensive educational programs are necessary to improve student and intern knowledge of gingival retraction advancements and side effects.

## Background:

Restoration and replacement of compromised or missing teeth are key objectives in dentistry. These can be accomplished sing fixed prostheses to restore function and aesthetics [1]. Fixed dental prosthesis is a general term for any prosthesis that is safely fixed to a specific tooth or teeth and cannot be removed by the patient [2]. Harmonious functioning of the neighboring soft and hard tissues with the fixed prosthesis requires precise duplication of the tooth preparation, finish line and gingival sulcus [3] and an accurate impression is crucial for creating successful long-lasting prostheses [4, 5 and 6]. Effective manipulation of the gingival tissue is crucial for obtaining an accurate dental impression, particularly in complex cases where the finish line is equigingival or sub gingival [6, 7, 8-9]. A sulcular width ≥0.2 mm is essential to prevent tearing of the impression material [10]. Gingival retraction is defined as displacement of the marginal gingiva away from the tooth [6, 9]. This generates vertical and lateral spaces essential for revealing subgingival margins and ensures sufficient bulk of the injected impression material in the gingival crevice. Moreover, gingival retraction aids in attaining hemostasis to guarantee adequate isolation for impression making using hydrophobic materials and during insertion of bonded restorations [5, 6]. The optimal gingival retraction technique should temporarily and a traumatically displace the gingiva to the greatest extent possible while ensuring sufficient hemostasis [5]. Gingival retraction can be accomplished using mechanical, chemo mechanical and surgical techniques such as electro surgery and rotational curettage [11]. The mechanical technique of gingival retraction using a plain retraction cord has been regularly practiced for several years. The cord mechanically displaces the gingiva from the finish line; however, its efficacy is limited by the lack of control over sulcular fluid exudation [12, 13]. Nevertheless, gingival sulcus expansion with management of fluid exudation from its walls can be effectively achieved through a combination of chemical action and pressure packing. The chemo-mechanical technique, which uses retraction cords soaked with hemostatic chemicals and astringents, is the most commonly used method. Chemicals used with retraction cords can be categorized as vasoconstrictors, such as epinephrine and astringents, such as aluminum chloride, aluminum potassium sulfate and ferric sulfate. Although surgical retraction techniques are rapid, they are detrimental and necessitate tissue resection [14]. In 2023, a survey conducted among dentists in Libya by Mahjoub *et al.* revealed that nearly all participants considered retraction cords essential for obtaining final impressions for permanent restorations [15]. In a recent study to determine the gingival retraction techniques used by private dentists in India, Altaf *et al.* found that 67% of doctor's used retraction cords for gingival retraction [16]. Therefore, it is of interest to identify the familiarity of dental interns and students at Saudi Arabia with the concept of gingival retraction during final impression making.

## Methods:

The study included all 4th and 5th year dental students and dental interns enrolled in the Bachelor of Dental Surgery program at Qassim University for the year 2023-2024 and the research procedure was approved by the Committee of Research Ethics of Qassim University (approval number: 23-55-03). Participants were selected based on the following inclusion and exclusion criteria. All eligible students and interns were invited to voluntarily complete an electronic questionnaire. Students who were not enrolled in 4thor 5th year or who had dropped or frozen their semesters were excluded. The aim and methodology of the study were explained to all participants and a questionnaire designed based on previous studies [15, 16, 17- 18] was administered via Google forms. Collected data consisted of demographic variables (gender, college campus, educational level) and knowledge/awareness items related to gingival displacement Data were analyzed using the SPSS software version 22 (IBM Corp; Armonk, NY, USA). All statistical tests were two-tailed, with P≤0.05. The overall knowledge and awareness regarding gingival retraction prior to making a final impression were assessed by summing up each awareness item which was scored as 1 for 'Yes' and 0 for 'No'. The total scores ranged from 0 to 15 (0-100%). Scores of ≥12 (≥80%) indicated high awareness, scores of ≥10 and ≤11 (67-73%) indicated moderate and scores of ≤9 (≤60%) indicated low awareness. Descriptive analyses were performed by recommended frequency distributions and percentages for the study variables, including participants' sex and academic level. In addition, participants' awareness and practice of gingival retraction prior to making a final impression were tabulated and the overall awareness level was plotted on a graph. The relationships of knowledge and practice with participants' sex and academic level were evaluated with Pearson's chi-square or exact probability tests based on the frequency distributions. This manuscript adheres to the STROBE guidelines [17].

## Results:

A total of 62 dental students and interns were included in this study. Of these, 44 (71%) were male and 18 (29%) were female. Regarding the academic level, 18 (29%) were in their 4th year, 25 (40.3%) were in their 5th year and 19 (30.6%) were interns (Table 1 - see PDF). Regarding participants' knowledge and awareness about gingival retraction before making final impressions, the highest agreement was reported for the necessity of gingival retraction cords for successful clinical practice (98.4%), followed by the intention to recommend gingival retraction to junior colleagues (95.2%). Furthermore, 90.3% of participants believed that the gingival sulcus recorded in the impression should be duplicated in the model, 66.1% considered cord packing challenging and 66.1% believed that patients may experience systemic complications after gingival displacement. The least reported agreement was regarding the correlation of retraction procedures with gingival recession (38.7%) and 40.3% of participants believed that a successful preparation and impression could be achieved without using are traction cord. Notably, male and female participants exhibited significant differences in agreement regarding the possibility of achieving successful preparations and impressions without using retraction cords (47.7% vs. 22.2%, respectively; P=0.049) and the likelihood of patients experiencing systemic complications after gingival displacement (56.8% vs. 88.9%, respectively; P=0.015) (Table 2 - see PDF). Regarding knowledge and awareness of gingival retraction prior to making a final impression according to the participants 'academic level, significantly differences were observed in only two items. First, 16.7%, 44% and 52.6% of 4th-year students, 5th-year students and interns, respectively, believed that retraction procedures could cause gingival recession (P=0.046). Second, 66.7%, 32% and 47.4% of 4th year students, 5th year students and interns, respectively, evaluated the pulse rate and blood pressure before applying retraction cords (P=0.049) (Table 3 - see PDF). Of the study participants, 30 (48.4%) and 11 (17.7%) participants had overall low and high knowledge and awareness regarding gingival retraction prior to making a final impression, respectively (Table 4 - see PDF). Furthermore, 22.2% and 15.9% of female and male students had high awareness levels, respectively and the difference was not statistically significant (P=.107). In addition, 27.8% of 4th-year students had high awareness levels compared with 12% of 5th-year student and 15.8% of interns, with no significant difference (P=501) (Table 5 - see PDF). Among participants practicing gingival retraction before making final impressions, 61 (98.4%) practiced fixed prosthodontics, 44 (71%) checked the patient's medical history before applying retraction cords and 33 (53.2%) wetted the retraction cord before removing it from the gingival sulcus.

## Discussion:

In this study, 98.4% of the participants agreed that gingival retraction cords were necessary for successful clinical practice. This is constant with the findings of Mahjoub & Elfallah that dental professionals used and believed that these tools were very important [15]. The findings that 95.2% of participants strongly wanted to advocate gingival retraction to junior colleagues and 90.3% of participants believed that the gingival sulcus recorded in the impression should be correctly duplicated in the model indicate good awareness of the clinical relevance of the procedure. Nevertheless, when examining the overall levels of knowledge and awareness, the study revealed that almost half of the participants (48.4%) had low levels of knowledge and awareness, whereas only 17.7% had high levels. Notably, statistical analysis revealed no significant difference in the overall knowledge and awareness levels between male and female participants, or between participants at different academic levels. This implies that both male and female students, as well as student's at all academic levels, have knowledge gaps. Although no significant differences in the overall knowledge and awareness levels were observed, our study revealed that the participants were aware of some elements of gingival retraction. For example, only 38.7% of participants believed that the retraction procedure could cause gingival recession. This is different from the findings of Abraham *et al.* found, that a 75% of practitioners knew that hemostatic agents could cause gingival damage [18]. This lack of understanding is particularly concerning because gingival recession is a major clinical problem. Female participants were more aware of the likelihood of patients experiencing systemic complications after gingival displacement than male participants and the difference was statistically significant. Participants' academic level considerably affected their awareness of certain complications. The belief that retraction treatments can lead to gingival recession increased from 16.7% in4th-year students to 44% in5th-year students and 52.6% in interns. Similarly, knowledge about measuring blood pressure and pulse rate before using retraction cords varied significantly depending on the academic level: 66.7% in 4th-year students, 32% in 5th-year students and 47.4% in interns. These specific item-level significant differences indicate that although the overall knowledge scores may not change, the depth of understanding of certain important aspects and possible complications changes significantly as students move up in school and between sexes. Thus, the null hypothesis is partially rejected. Another essential practical aspect examined was the wetting of retraction cords before removal. Removal of a dry cord may injure the delicate epithelial lining, highlighting the importance of this practice. In this study, 53.2% of the participants wetted the retraction cords. This is similar to the findings of Reddy *et al.* (69.2%) [19]. But different from those of Donovan *et al.* (33.94%) [5]. the fact that our overall results are consistent and reveal specific knowledge gaps supports the need for ongoing professional development.

The disparities in specific knowledge items based on sedan academic level indicate the feasibility of targeted educational interventions. Particularly, the low awareness regarding gingival recession as a complication of gingival retraction in all groups and the differences in awareness regarding systemic complications and pre-procedural checks highlight the need for more attention to these important topics in the curriculum. This study had several limitations. First, because cross-sectional studies only record data at a particular moment in time, establishing cause-and-effect relationships or monitoring changes in knowledge and awareness over an extended period was impossible. Second, the use of self-reported data gathered via a questionnaire introduces the possibility of response bias, because participants may have overestimated their knowledge or provided socially acceptable answers. Third, because the study only included dental students and interns at one university, the generalizability of the results to other Saudi Arabian universities, regions, or larger international contexts is limited by differences in curricula, teaching styles and clinical exposure. Finally, the comparatively smaller sample size may have inadequate statistical power to recognize more understated differences or associations, which would further affect the wider applicability of the results, although they represent a portion of the institution's target population. Several strategies could be used in future studies to overcome these limitations. First, longitudinal designs could be used to overcome the limitations of cross-sectional designs. This would entail following the same group of students through various academic stages to monitor how their understanding, consciousness and practical abilities in gingival retraction develop over time and to evaluate the effects of curriculum modifications or educational interventions. Second, future studies could use objective evaluation techniques to reduce the inherent bias in self-reported data. This could involve direct observations of clinical procedures by knowledgeable faculty members, objective structured clinical examinations, or practical examinations. These techniques would eliminate any potential disparities between perceived and actual knowledge and practice and offer a more accurate assessment of actual clinical competence. Third, a multicenter design might improve the generalizability of the findings. Working together with other dental schools in Saudi Arabia or abroad would ensure more varied participant pool, with differences in clinical exposure, teaching philosophies and curricula, thereby enhancing the external validity of the results. Finally, future studies should aim for larger and more statistically robust samples. To improve the reliability and statistical power of the results, a power analysis could be performed before the study to ascertain the ideal sample size required to confidently identify significant differences and associations. According to Ansari *et al.*'s research, dental students in Riyadh City strongly prefer a specific material for gingival retraction alternatives or primary or secondary impressions [20]. Elsayed *et al.* found that although the majority of Saudi Arabian dentists gave PFDPs to their patients, there are still a lot of unanswered questions, especially about the materials and methods used for manufacture [21]. This study on dental interns in the Qassim region of Saudi Arabia showed no statistically considerable variation in the knowledge and awareness levels regarding fixed Prosthodontics and gingival retraction method. However, there is a considerable requisition to improve expertise through continuous education to improve awareness of modern, advanced gingival retraction techniques. This study helps to create awareness among the interns to improve the knowledge.

## Conclusion:

We found no statistically considerable variation in the knowledge and awareness levels of dental students and interns Saudi Arabia, based on sex or academic level. However, some major gaps in knowledge regarding possible complications and the need for tests before the procedure were identified. Accordingly, the null hypothesis was partially rejected. Thus, we show the need for targeted educational programs and full courses to help students and interns strengthen their knowledge regarding new methods of gingival retraction and the possible side effects and complications associated with these procedures.

## Author contribution:

Conceptualization, Rawan Alrethia and Ibrahim Albaqami; Formal analysis, Rawan Alrethia, Ibrahim Albaqami, Abdulaziz Alharbi and Mohammed Alqahtani; Methodology, Rawan Alrethia, Ibrahim Albaqami and Bayan Almohaimeed; Resources, Rawan Alrethia, Ibrahim Albaqami, Bayan Almohaimeed,Abdulaziz Alharbi and Mohammed Alqahtani; Writing -Original Draft, Rawan Alrethia, Ibrahim Albaqami, Bayan Almohaimeed, Abdulaziz Alharbi and Mohammed Alqahtani; Writing -Review & Editing, Rawan Alrethia, Ibrahim Albaqami, Bayan Almohaimeed,Abdulaziz Alharbi and Mohammed Alqahtani.

## Availability of data and materials:

The datasets analysed in this study are freely provided.

## Financial support and sponsorship:

Self

## Competing Interests:

The authors have no conflicts of interests to declare.

## References

[1] Sailer I (2022). Periodontol 2000..

[2] Ferro KJ (2017). J Prosthet Dent..

[3] Chauhan R (2025). World J Methodol..

[4] Wang Y (2019). Quintessence Int..

[5] Donovan TE, Chee WW (2004). Dent Clin North Am..

[6] Al Hamad KQ (2008). J Clin Periodontol..

[7] Martins FV (2021). J Prosthet Dent..

[8] Donovan TE, Chee WW (2004). Dent Clin North Am..

[9] Safari S (2016). J Dent Biomater..

[10] Laufer B-Z (1996). J Prosthet Dent..

[11] Benson BW (1986). J Prosthet Dent..

[12] Harrison J (1961). J Prosthet Dent..

[13] Woycheshin FF (1964). J Prosthet Dent..

[14] Donovan TE (1985). J Prosthet Dent..

[15] Mahjoub HE, Elfallah KI (2023). International Journal of Science and Research (IJSR)..

[16] https://www.oraljournal.com/archives/2020/vol6issue1/PartD/8-3-13-116.pdf.

[17] Von Elm E (2007). BMJ..

[18] Abraham IA (2023). BMC Oral Health..

[19] Reddy SG (2016). Journal of or facial Sciences..

[20] Ansari AS (2021). J Family Med Prim Care..

[21] Elsayed HM (2025). Prosthesis..

